# An Integrated Diagnostic Approach to Severe Generalized Canine Tetanus: Serial Neurological, Clinicopathological and Electromyographic Assessment with Successful Clinical Management

**DOI:** 10.3390/vetsci13090886

**Published:** 2026-08-30

**Authors:** Andrei Răzvan Codea, Amalia Marina Neagu, Ștefania-Mădălina Dandea, Helga Demény, Bogdan Florea, Yarden Lamy Casoy, Mihaela Niculae

**Affiliations:** 1 Department of Internal Medicine, University of Agricultural Sciences and Veterinary Medicine, Calea Mănăștur 3–5, 400372 Cluj-Napoca, Romania; razvan.codea@usamvcluj.ro (A.R.C.); stefania.dandea@usamvcluj.ro (Ș.-M.D.); 2 Emergency Hospital, University of Agricultural Sciences and Veterinary Medicine, Calea Mănăștur 3–5, 400372 Cluj-Napoca, Romania; amalia.neagu@usamvcluj.ro; 3 Demed Vet, Piața 1848 No. 2, 400058 Cluj-Napoca, Romania; 4 Epilepsy and EEG Monitoring Center, 400696 Cluj-Napoca, Romania; consult@epilepsie-eeg.ro; 5 Regina Maria Medical Center, 400696 Cluj-Napoca, Romania; 6 Veterinary Teaching Hospital, Koret School of Veterinary Medicine, The Hebrew University of Jerusalem, P.O. Box 12, Rehovot 761001, Israel; yardenlamy@gmail.com; 7 Knowledge Farm Veterinary Specialists Referral Centre, Kfar Shaba 4442404, Israel; 8 Department of Infectious Diseases, University of Agricultural Sciences and Veterinary Medicine, Calea Mănăștur 3–5, 400372 Cluj-Napoca, Romania

**Keywords:** antitoxin antibodies, canine tetanus, clinicopathological assessment, diagnostic, electromyography, intensive care, neurological examination

## Abstract

Tetanus is uncommon in dogs, which are considered relatively resistant to tetanospasmin, and detailed descriptions of the diagnostic evaluation of severe generalized cases remain limited. A 5.5-month-old intact male Miniature Schnauzer was presented four days after a puncture wound to the right pelvic limb. On admission the dog was alert but non-ambulatory, with generalized muscle rigidity, tremors, marked cervical hyperextension, hyperactive spinal reflexes, facial muscle spasms and dysphagia. Together with the wound history and rapid progression, these findings supported a presumptive diagnosis of generalized tetanus, clinically classified as Grade III. Serum collected before tetanus antitoxin administration contained no detectable antibodies against *Clostridium tetani* neurotoxin. Treatment comprised equine tetanus antitoxin, antimicrobials, multimodal sedation and muscle relaxation, enteral nutrition, intensive nursing and physiotherapy. Needle electromyography on day 10 showed increased insertional activity, positive sharp waves and fibrillation potentials, and continuous spontaneous motor unit activity. Despite patient young age and rapid disease progression, clinical improvement was evident from day 10, and the dog was discharged on day 16, ambulatory and eating voluntarily. This case highlights that severe generalized canine tetanus remains principally a clinical diagnosis, while serial neurological examination, clinicopathological monitoring and electromyography can strengthen diagnostic confidence, characterize disease progression and support clinical management.

## Simple Summary

Tetanus is an uncommon but potentially life-threatening disease in dogs caused by a toxin produced by the bacterium *Clostridium tetani*. This case report describes a 5.5-month-old Miniature Schnauzer that developed severe generalized muscle stiffness, muscle spasms, difficulty swallowing and inability to walk four days after a puncture wound of a hind limb. The diagnosis was based on the wound history, characteristic clinical signs, repeated neurological examinations, blood tests and electrical testing of muscle activity. A blood sample taken before treatment showed no detectable antibodies against the tetanus toxin. Despite features associated with a poorer prognosis, including patient young age and rapid and severe progression of the disease, the dog responded to intensive treatment including tetanus antitoxin, antibiotics, medications to reduce muscle spasms and discomfort, nutritional support, nursing care, and physiotherapy. The dog showed marked clinical improvement and was discharged after 16 days. This case illustrates the importance of recognizing the characteristic signs of tetanus early and combining repeated clinical assessment with supportive diagnostic tests and intensive care. Further studies involving larger numbers of dogs are needed to improve prognostic assessment and determine how electrical testing of muscle activity may contribute to diagnosis and monitoring.

## 1. Introduction

Tetanus is an acute, non-contagious neurological disease caused by tetanospasmin, a potent neurotoxin produced by *Clostridium tetani*, an obligate anaerobic, spore-forming bacterium widely distributed in soil and the gastrointestinal tract of animals [1]. Infection most commonly occurs following the contamination of open wounds with bacterial spores. The presence of devitalized or necrotic tissues creates the anaerobic environment that promotes spore germination, proliferation of vegetative forms and the consequent tetanus neurotoxin (TeNT) production during the late exponential growth phase [2]. Once released, tetanospasmin binds irreversibly to peripheral motor nerve terminals at the neuromuscular junction, where it is internalized by receptor-mediated endocytosis and undergoes retrograde axonal transport to the central nervous system (spinal cord and brain), followed by trans-synaptic transfer to inhibitory interneurons [1,3,4]. The light chain of TeNT acts as a zinc-dependent endopeptidase and cleaves synaptobrevin/vesicle-associated membrane protein (VAMP), a component of the soluble N-ethylmaleimide-sensitive factor attachment protein receptor (SNARE) complex required for synaptic vesicle fusion [1,5,6]. This prevents vesicular release of the inhibitory neurotransmitters glycine and γ-aminobutyric acid (GABA), resulting in loss of inhibitory control over motor neurons and persistent motor neuron activity [1,3,5,6]. The consequent loss of glycinergic and, to a lesser extent, GABAergic inhibitory interneuron activity explains tetanus’ characteristic clinical picture that includes sustained muscle contraction, generalized rigidity, hyperreflexia, and painful muscle spasms [1,6,7,8].

Among the susceptible hosts, domestic carnivores appear to exhibit a relative natural resistance to tetanus compared with horses and humans, and consequently the disease is considered uncommon or rare in small animal practice [1,8]. Nevertheless, generalized tetanus in companion animals remains a life-threatening neurological emergency associated with substantial morbidity and prolonged hospitalization [9].

Clinical presentation in cats and dogs varies according to the extent of neuronal involvement and is generally classified as localized or generalized disease. Relatively restricted neuronal involvement is associated with localized muscle rigidity near the site of toxin entry, whereas more extensive involvement of spinal and brainstem inhibitory networks is associated with generalized disease [1,3,4,8]. Localized tetanus is relatively more common in cats [8,10,11,12,13,14,15] and manifests as muscle stiffness confined to a single limb or body region and may progress to the generalized form. Generalized tetanus is the most frequently recognized presentation in dogs [8,9,16,17,18,19,20,21,22,23] and is characterized by diffuse muscle rigidity, extensor limb stiffness, facial abnormalities (trismus, prolapsed third eyelids, risus sardonicus), erect ear carriage, cervical extension, dysphagia, ptyalism, regurgitation, vomiting, trembling, hyperesthesia, and generalized hyperreflexia [8]. Other clinical signs include torticollis, urethral and anal sphincter hypertonicity, ataxia, spastic tetraplegia, anorexia and lethargy [17]. In severe cases, respiratory compromise secondary to laryngospasm or respiratory muscle rigidity, autonomic dysfunction, aspiration pneumonia, as well as musculoskeletal complications due to sustained muscle contraction were reported [1,10,22,24].

A severity classification system for canine tetanus was proposed by Burkitt et al. [20], adapted from the scale used in human medicine. Class I comprises dogs with facial signs of tetanus; class II dogs with dysphagia and generalized rigidity, with or without facial signs; class III dogs with class I and/or class II signs together with recumbency and seizures; and class IV dogs with the preceding signs together with abnormal heart rate or rhythm, abnormal arterial blood pressure or abnormal respiration. The scheme has subsequently been applied, in the original or in a modified form, in canine and feline reports [9,16], and provides a practical framework for describing disease severity at presentation.

Neurological abnormalities associated with generalized tetanus may extend beyond generalized muscle rigidity. Cranial nerve dysfunction, impaired postural reactions, altered gait, and multifocal neurological deficits have been described in severe canine cases, reflecting the widespread effects of tetanospasmin within the central nervous system [21]. In addition, autonomic disturbances and sleep disorders, including rapid eye movement (REM) sleep behavior disorder, have recently been recognized as part of the neurological spectrum of canine tetanus, emphasizing that the disease affects multiple functional neural networks rather than the motor system alone [25,26,27].

Although tetanus is primarily a clinical diagnosis based on the characteristic neurological signs and a compatible history of wound contamination, establishing the diagnosis may be challenging during the early stages of disease or in patients with atypical presentations [1,8]. Isolation of *C. tetani* from a suspected wound has low diagnostic sensitivity, and TeNT detection in biological samples is rarely successful because very small quantities of toxin can produce clinical disease [1,8]. Consequently, laboratory confirmation is not routinely achievable, and negative microbiological or toxin-detection results do not exclude tetanus [1,8]. Ancillary investigations are therefore mainly useful for excluding competing neurological or neuromuscular disorders, assessing disease severity, identifying complications, and supporting clinical decision-making [8,9]. A structured diagnostic approach integrating history, serial neurological examination, clinicopathological assessment, and, when indicated and available, electrodiagnostic testing may increase diagnostic confidence, facilitate early recognition, and support clinical management.

Treatment of canine tetanus relies on a multimodal approach aimed at neutralizing circulating toxin, eliminating the source of infection, controlling muscle rigidity and spasms, minimizing external stimulation, maintaining adequate nutrition and hydration, and preventing secondary complications. Intensive nursing care, physiotherapy, enteral nutritional support, and careful monitoring of respiratory and autonomic function are nowadays recognized as fundamental components of successful management [8,16,28,29]. Recent retrospective studies have reported survival rates ranging from approximately 76% to 89% in dogs receiving intensive supportive treatment, reflecting substantial improvements in critical care and standardized therapeutic protocols [9]. Nevertheless, recovery is frequently prolonged, and neurological deficits may evolve throughout hospitalization, necessitating repeated neurological assessment to guide clinical decision-making and monitor response to treatment.

Although previous retrospective studies and isolated case reports have substantially expanded current knowledge of the clinical presentation, prognostic indicators, complications, and therapeutic management of canine tetanus [9,18,19,20], recent reports describing cranial nerve dysfunction, advanced imaging findings, and rapid eye movement (REM) sleep behavior disorder have further broadened the recognized neurological spectrum of the disease [21,27]. However, reports focusing on the diagnostic pathway, including the integration of serial neurological examinations, clinicopathological findings, and electrodiagnostic testing in the diagnosis and monitoring of generalized tetanus, remain scarce.

The present case report describes a juvenile dog with generalized tetanus clinically classified as grade III based on the information available at presentation, emphasizing the diagnostic reasoning that led to the clinical diagnosis and the contribution of serial neurological examinations, clinicopathological monitoring, and needle electromyography to disease characterization and clinical management. In addition, the absence of detectable circulating antitoxin antibodies was documented before passive immunization. Despite several recognized negative prognostic indicators, the patient was discharged ambulatory after 16 days. Finally, aspects of the therapeutic protocol that differed from current recommendations are reported transparently and discussed in the context of this case.

## 2. Case Description

A 5.5-month-old intact male Miniature Schnauzer weighing 7.3 kg was referred to the Veterinary Emergency and Critical Care Hospital of the Faculty of Veterinary Medicine Cluj-Napoca, Romania with a presumptive diagnosis of poisoning, specifically suspected metaldehyde intoxication. According to the anamnesis, four days prior to the clinic presentation, the animal sustained a puncture wound to the right pelvic limb, followed by progressive lameness for approximately two days. Within the 24 h preceding referral, the dog developed generalized muscle stiffness and difficulty prehending food.

**Figure 1:**
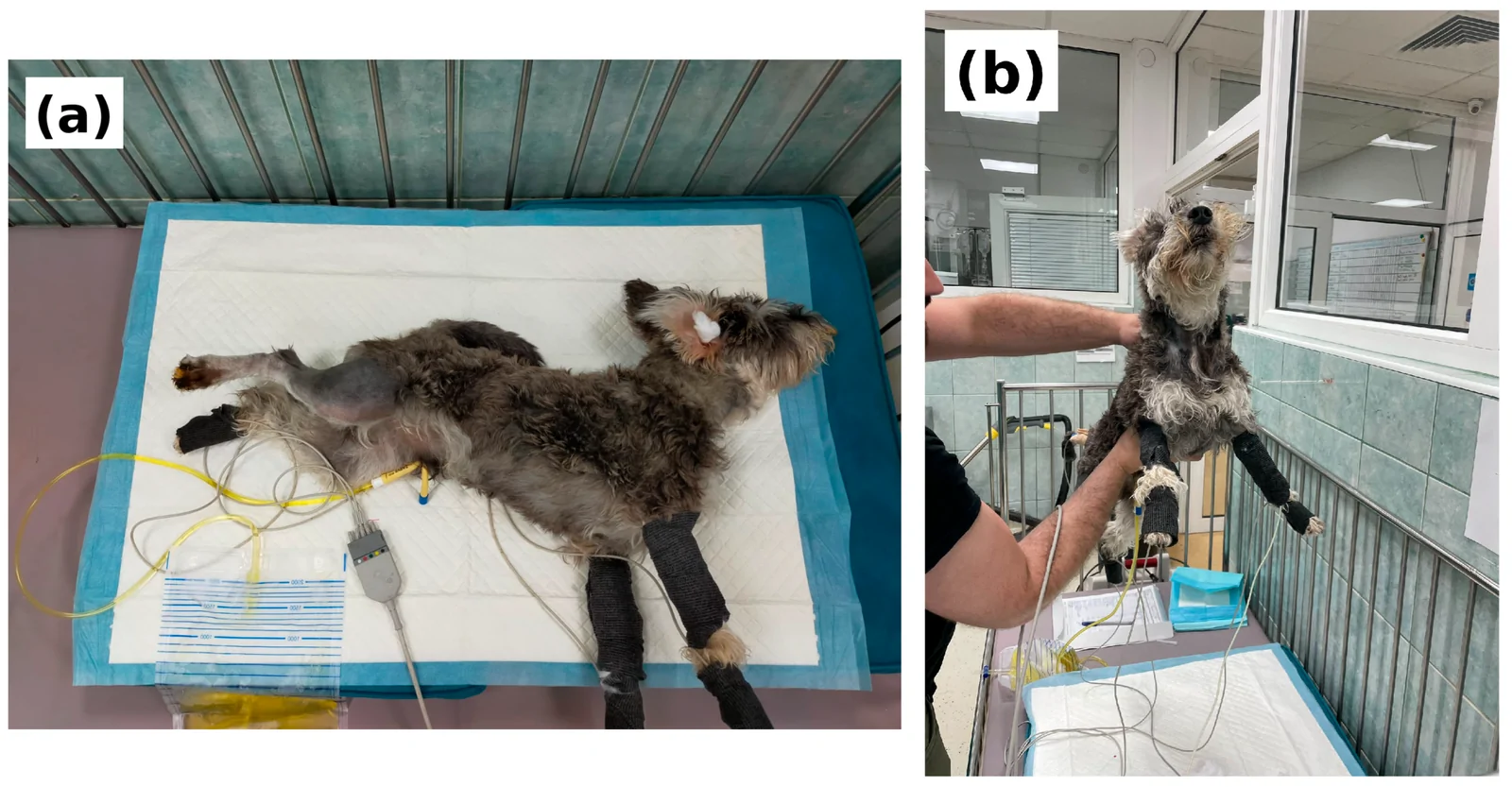
Clinical appearance of the dog on the day of admission (day 1). (**a**) Patient in lateral recumbency, showing marked cervical hyperextension, rigid thoracic limb extension with generalized muscular hypertonicity and rigidity, a urinary catheter and the electrocardiographic leads used for monitoring; (**b**) Patient supported in a standing position by an operator, showing marked cervical hyperextension and rigid extension of the thoracic limbs.

**Figure 2:**
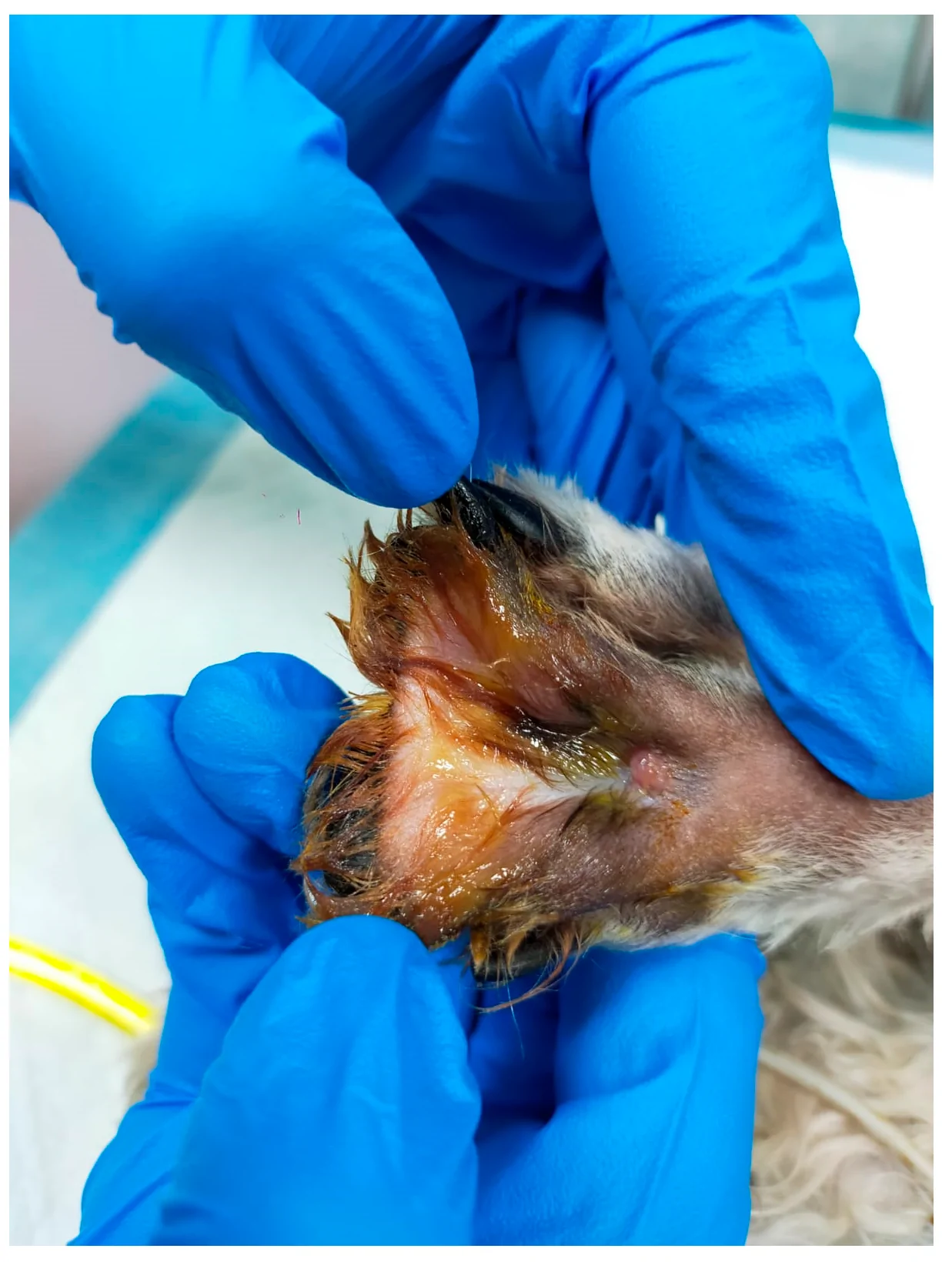
Puncture wound in the interdigital space of the right pelvic limb, identified on admission at the site indicated by the owner. The lesion was closed, without devitalized or necrotic tissue and without discharge; the discoloration of the surrounding hair is due to the antiseptic applied during examination.

On admission, the dog underwent a general clinical and paraclinical examination. The patient was conscious, normothermic (38.7 °C), but non-ambulatory as he exhibited generalized muscle rigidity, tremors, and marked cervical hyperextension. Flexion and extension of all limbs were severely restricted due to generalized hypertonicity. The clinical appearance of the patient on admission is shown in Figure 1. A small puncture wound was identified in the interdigital space of the right pelvic limb, at the site pointed out by the owner. The lesion was closed, with no devitalized or necrotic tissue and no discharge, and surgical debridement was therefore not considered necessary. The appearance of the wound is shown in Figure 2. No sample was submitted for bacteriological culture. Complete blood count, serum biochemistry, blood gas analysis, and urinary catheterization were also performed.

A serum sample was additionally submitted to Laboklin GmbH & Co. KG (Bad Kissingen, Germany) for assessment of antibodies against tetanus neurotoxin. The sample was collected on day 1, within the hour preceding the first intravenous dose of tetanus antitoxin and therefore represented a pre-treatment sample; the laboratory report (no. 2206-R-35553) was issued six days later, on day 7 of hospitalization. Following retrospective clarification obtained directly from LABOKLIN, antibody testing was confirmed to have been performed using a lateral-flow assay (LFA) by an external accredited veterinary diagnostic laboratory on behalf of LABOKLIN. Results were reported semi-quantitatively as negative (−), +, ++, or +++, and the pre-treatment sample was reported as negative (−). As anti-tetanus serology reflects pre-existing humoral immunity rather than confirming acute tetanus, this result was considered supportive information and was not used to establish the diagnosis. Following direct inquiry, LABOKLIN confirmed that the manufacturer, catalogue/reference number, and assay-specific analytical and validation characteristics could not be retrieved retrospectively from the available documentation. Consequently, an assay-specific quantitative cut-off or limit of detection cannot be reported for this historical result.

Neurological examination revealed an alert and responsive dog with a normal mental status. Generalized muscle hypertonicity produced a rigid body posture with markedly restricted limb movement, which affected the dog’s ability to maintain balance. Cranial nerve abnormalities were limited to facial muscle spasms and dysphagia, whereas eye movements remained normal. Spinal reflexes were hyperactive throughout the body. Responses to nociceptive stimulation appeared reduced during this initial examination, although their interpretation was substantially limited by the marked generalized rigidity. Postural reactions could not be assessed reliably because of the severe rigidity. Overall, the neurological findings suggested multifocal involvement.

Taking into account the history of a recent puncture wound, the rapidly progressive generalized muscle rigidity, the facial muscle spasms, the dysphagia and the preserved mentation, generalized tetanus was considered the primary diagnostic suspicion. Based on the facial muscle spasms, dysphagia, generalized rigidity and non-ambulatory status, the presentation was clinically classified as grade III generalized tetanus according to the published severity classification [20], the grade being assigned on the basis of recumbency, as in previous reports that applied the scheme in a modified form [9,16]. Generalized tetanic spasms were present throughout the acute phase; no epileptic seizures were documented at any time during hospitalization, and the tetanic spasms were not interpreted as epileptic events. Because heart rate, cardiac rhythm and arterial blood pressure were not recorded at admission, autonomic dysfunction could not be formally excluded, and class IV disease cannot be ruled out retrospectively.

The principal differential diagnoses included strychnine intoxication, inflammatory myopathy, meningoencephalitis, cervical spinal cord disease, and metabolic causes of generalized neuromuscular hyperexcitability, particularly hypocalcemia [1,28,30]. Strychnine intoxication was identified as an important differential because antagonism of inhibitory glycinergic neurotransmission can produce severe stimulus-sensitive extensor rigidity and spasms closely resembling generalized tetanus [1,28,30]. In contrast, the preceding puncture wound, progressive evolution of clinical signs over several days, dysphagia, and facial muscle involvement, together with the absence of a known toxic exposure, favored tetanus. Inflammatory myopathy was included as well because generalized muscle stiffness may accompany primary muscle disease and the early manifestations of tetanus may resemble a myopathy [1,28]. However, the pronounced hyperreflexia and stimulus-sensitive spasms, together with the clinical progression, were less consistent with a primary myopathy. Meningoencephalitis was also considered; still, the combination of preserved mentation, generalized rigidity, stimulus-sensitive spasms, hyperreflexia, dysphagia, and characteristic facial involvement was more supportive of tetanus [1,28,30]. Cervical spinal cord disease was evaluated separately because of the marked appendicular motor abnormalities, but a single cervical spinal cord lesion was considered less likely to account for the combination of generalized muscle rigidity and spasms with facial involvement and dysphagia [1,28]. Metabolic tetany due to hypocalcemia, characterized by increased neuromuscular excitability associated with decreased ionized calcium [1,28], was not supported by the normal total and ionized calcium concentrations in this dog.

Metaldehyde intoxication, which was the presumptive diagnosis at referral, was also evaluated because it can produce signs of marked central nervous system excitation, including tremors, hyperesthesia, hyperthermia, and seizures [28,31,32]. In canine metaldehyde intoxication, clinical signs typically develop rapidly following ingestion [31,32]. In contrast, the progressive evolution of signs over several days in the present case, together with the absence of a known exposure and the presence of a preceding puncture wound, dysphagia, and facial muscle involvement, made this diagnosis less likely.

Diagnostic investigations were therefore directed toward supporting the clinical diagnosis, excluding alternative disorders, and establishing baseline clinicopathological parameters for patient monitoring.

Serial neurological reevaluations were conducted throughout the hospitalization period to monitor the patient’s response to therapy and progression of neurological recovery. The clinical course was unfavorable during the first days of hospitalization, with worsened muscle rigidity and development of opisthotonus and vocalization on day 2 and day 5, respectively. The first clear signs of improvement as response to the multimodal treatment protocol were observed on day 10. Thereafter, muscle tone gradually normalized, rigidity diminished and the dog’s posture gradually improved. Hyperactive spinal reflexes observed during the initial neurological examination returned within expected physiological limits, exaggerated responses to external stimuli subsided as a positive response to sedation, muscle relaxants, antimicrobial therapy, and supportive care. During serial examinations throughout the 16-day hospitalization period, responses to nociceptive stimulation became more consistent and appropriate as the patient’s clinical condition improved. This finding suggested progressive resolution of the neurological dysfunction associated with tetanospasmin activity. Postural reactions also improved; impaired balance and difficulty maintaining equilibrium progressively resolved, and the dog demonstrated improved coordination and stability during standing and ambulation. The ability to correct posture when gently displaced returned progressively, further supporting neurological recovery and favorable therapeutic response. By day 16, voluntary ambulation and food intake, as well as normal postural function had substantially improved.

**Figure 3:**
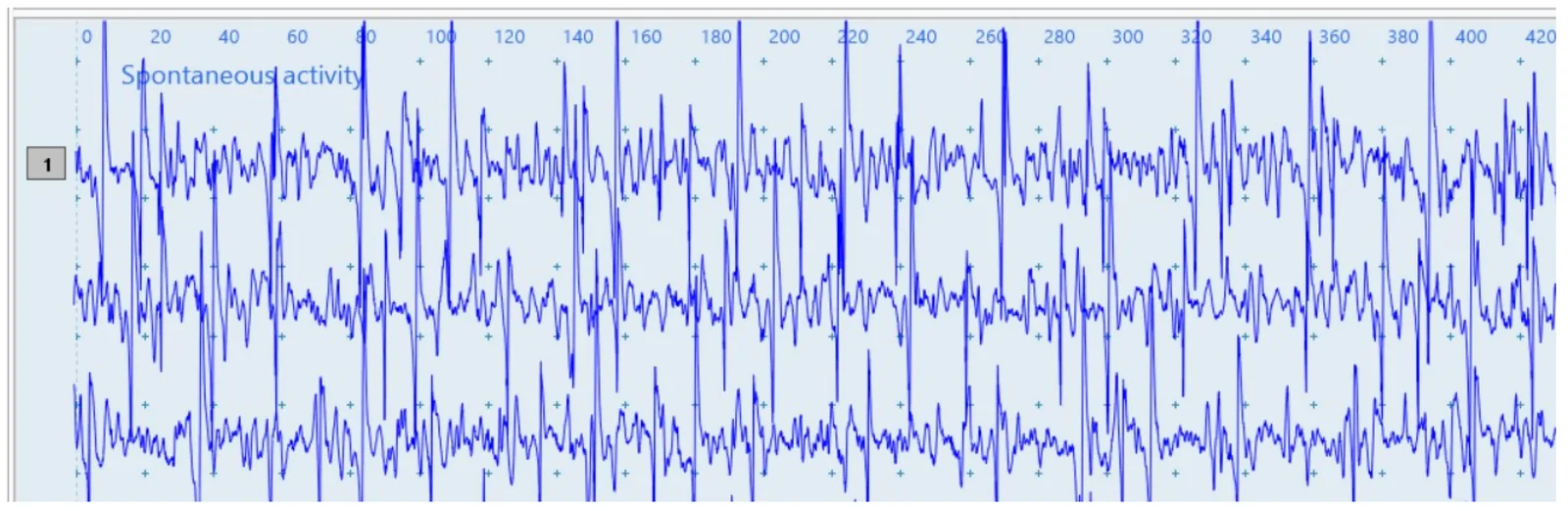
Representative needle electromyographic recording obtained on day 10 of hospitalization, under general anesthesia, showing pathological spontaneous activity (positive sharp waves and fibrillation potentials) together with motor unit action potentials of variable morphology. Similar abnormalities were recorded in all five muscles examined, although they appeared less abundant in the distal muscles. Recording settings: low-frequency filter 20 Hz, high-frequency filter 5000 Hz, sweep speed 20 ms/division, sensitivity 250 µV/division.

Needle electromyography (EMG) was performed on day 10 of hospitalization under general anesthesia, with the dog positioned in left lateral recumbency. The trapezius, triceps brachii, epaxial musculature (erector spinae) at the level of the second lumbar vertebra, vastus medialis and tibialis cranialis muscles were examined. Only the muscles of the right side were sampled, as the dog remained in left lateral recumbency throughout the procedure and was not repositioned. All examined muscles showed moderately increased insertional activity and pathological spontaneous activity consisting of positive sharp waves and fibrillation potentials, together with continuous spontaneous motor unit activity characterized by repetitive motor unit action potentials of variable morphology and normal duration. On qualitative assessment, these abnormalities appeared more prominent in the proximal than in the distal muscles examined. As the examination was not performed prospectively using a standardized semi-quantitative grading system, and because retrospective review of the archived recordings did not permit reliable distinction of spontaneous activity of endplate origin from pathological spontaneous activity or reliable grading of individual muscles, this apparent proximal predominance should be regarded as a qualitative and exploratory observation rather than evidence of a true proximal-to-distal gradient. Recruitment and maximal contraction could not be assessed, as the recording was obtained under general anesthesia. The electromyographic pattern was considered consistent with generalized tetanus and, together with the history and the clinical findings, supported the diagnosis. A representative recording is shown in Figure 3.

Anesthesia for the procedure consisted of midazolam (0.2 mg/kg IV) and butorphanol (0.2 mg/kg IV) as premedication, followed by induction with propofol (3 mg/kg IV, titrated to effect). The trachea was intubated, and anesthesia was maintained with isoflurane in oxygen at an end-tidal concentration of 0.8 to 1.2%, adjusted according to anesthetic depth. Monitoring throughout the procedure comprised electrocardiography, non-invasive blood pressure measurement, pulse oximetry, capnography and body temperature. Core temperature remained within normal limits throughout the recording. No anesthetic complications and no exacerbation of tetanic spasms were observed.

Recordings were obtained with a Neuro-MEP-Micro electromyograph and its dedicated acquisition software (version 2009; Neurosoft Ltd., Ivanovo, Russia), using disposable concentric needle electrodes (model GA35A77, 35 × 0.45 mm, 26 G; SEI EMG s.r.l., Cittadella, Italy). The low-frequency filter was set at 20 Hz and the high-frequency filter at 5000 Hz, the sweep speed at 20 ms/division and the amplitude scale at 250 µV/division. The electromyograph and the accompanying software are intended for use in human patients and no canine reference database is implemented. The automated quantitative analysis module was not used because no canine reference values are available, and its automatic classification of the recordings was not considered in the interpretation of the study. For this reason, the quantitative output of the instrument, including motor unit potential amplitude and duration norms and the automated classification of the recordings, was not used, and the examination was interpreted qualitatively by the two veterinary neurologists who performed the examination. The nerve and spinal root labels automatically generated by the software were not reported because they correspond to human anatomy rather than canine anatomy.

Hematology was performed on days 1 and 4, serum biochemistry on days 1, 2 and 9, and venous blood gas analysis on days 1, 2 and 4. All blood gas samples were obtained by jugular venipuncture. Serum biochemistry results are presented in Table 1, blood gas results in Table 2 and haematological results in Table 3. On day 2, blood was additionally collected for Canine C-reactive protein (CRP): 18.2 mg/L (<20 mg/L = normal; 20–30 mg/L = equivocal; >30 mg/L = abnormal) and D-dimer: 0.1 µg/mL (<0.3 µg/mL = normal; >0.3 µg/mL = abnormal). Basal serum cortisol measured on the same day was 18.9 ng/mL (reference interval 15 to 65 ng/mL).

Analyses performed on day 1 were carried out in house on a VetScan VS2 dry chemistry analyzer (Abaxis Inc., a Zoetis company, Union City, CA, USA). Analyses performed on days 2 and 9 were carried out at the laboratory of the Discipline of Pathology and Medical Clinic, Faculty of Veterinary Medicine, Cluj-Napoca, Romania, on a Screen Master Touch spectrophotometer (Hospitex Diagnostics, Sesto Fiorentino, Italy) for the biochemical and turbidimetric determinations. Haptoglobin was measured with a commercial reagent (ref. SWPH050HP, Swiss Pharm Import-Export, Codlea, Brasov, Romania). Fibrinogen was measured on an Amelung KC 1A ball coagulometer (Heinrich Amelung GmbH, Lemgo, Germany). Cortisol was measured with a commercial enzyme-linked immunosorbent assay (Cortisol ELISA, ref. DKO001, DIA.METRA S.r.l., Via Pozzuolo 14, 06038 Spello, Perugia, Italy). Reference intervals differ between the two laboratories and are those of the laboratory that produced each result. The exception is total protein, for which the canine reference interval of the Merck Veterinary Manual (Serum Biochemical Analysis Reference Ranges, https://www.msdvetmanual.com/multimedia/table/serum-biochemical-analysis-reference-ranges, accessed on 8 August 2026) is given, because the interval supplied with the analysis was outside the range reported for this species. Total globulins were measured only on day 1 (2.2 g/dL, reference interval 2.1 to 3.7 g/dL); the values reported on days 2 and 9 are γ-globulins.

All samples were obtained by jugular venipuncture. The analyser does not provide species-specific reference intervals for these parameters, and values are therefore reported without reference ranges. Haematocrit and total haemoglobin measured by the blood gas analyser differ slightly from the corresponding values obtained with the haematology analyser (Table 3), because the two instruments use different measurement principles.

Complete blood counts were performed on a VetScan HM5 hematology analyzer (Abaxis Inc., a Zoetis company, Union City, CA, USA) at the Laboratory of Hematology, Discipline of Physiopathology, Faculty of Veterinary Medicine, Cluj-Napoca, Romania. On day 4 the blood smear examination reported segmented neutrophils and reactive lymphocytes, a microhematocrit of 29% and a reticulocyte count of 0%, consistent with normocytic non-regenerative anemia and eosinopenia.

On admission (day 1), a urinary catheter was placed and treatment was started with lactated Ringer solution as a constant rate infusion (5 mL/kg/h) supplemented with a vitamin and amino acid solution (Duphalyte, 100 mL; Zoetis Belgium S.A., Louvain-la-Neuve, Belgium), together with equine tetanus antitoxin (Ser Clostetan, immunoserum tetanicum equinum nativum, minimum 300 IU/mL; Bioveta a.s., Komenskeho 212, 683 23 Ivanovice na Hane, Czech Republic) 70 mL, corresponding to 21,000 IU, intravenously every 24 h. Antimicrobial therapy consisted of amoxicillin with clavulanic acid 20 mg/kg every 12 h and metronidazole 20 mg/kg every 12 h. Multimodal management of the severe generalized rigidity and stimulus-induced tetanic spasms included levetiracetam 40 mg/kg every 12 h and diazepam 0.1 mg/kg every 8 h, together with butorphanol 0.1 mg/kg, ketamine 2 mg/kg intramuscularly, and methadone 0.5 mg/kg every 6 h for analgesic and sedative support. Levetiracetam was administered empirically as an adjunct to the control of rigidity and spasms and not for suspected or documented epileptic activity. Pantoprazole 1 mg/kg every 24 h and maropitant 1 mg/kg were administered as supportive treatment. Dexamethasone 0.2 mg/kg and hydrocortisone were administered mainly as premedication before the first intravenous administration of heterologous equine antitoxin.

On day 2, the clinical course was unfavorable, with accentuation of the muscle contractures and spasms and the appearance of opisthotonus. A peripherally inserted central catheter was placed in the right pelvic limb and a nasogastric tube was placed for enteral feeding. Phenobarbital 6 mg/kg intramuscularly every 12 h was administered as an adjunctive sedative and spasm-control medication in the context of worsening generalized muscle contractures and stimulus-induced spasms; it was not administered for suspected or documented epileptic seizures. Gabapentin 300 mg (41 mg/kg) by nasogastric tube every 12 h, metamizole sodium (Novasul 500 mg/mL; Vetviva Richter GmbH, Wels, Austria) 25 mg/kg every 12 h, tolperisone 20 mg/kg by nasogastric tube every 8 h and acepromazine 0.05 mg/kg were added. From this day the patient was repositioned every 4 h and fed by tube every 6 h.

On day 3, the supportive and symptomatic treatment was intensified because the clinical signs persisted. Oxygen therapy was instituted, and ketamine 0.5 mg/kg were administered intravenously as an additional component of the multimodal control of muscle rigidity and spasms. Hydrocortisone was repeated intravenously on empirical grounds. Baclofen (Lioresal 10 mg tablets; Novartis Pharma GmbH, Nuremberg, Germany) 0.7 mg/kg by introduced by nasogastric tube every 8 h as an additional muscle relaxant, together with a pyridoxine and aspartic acid solution (Aspatofort, Terapia S.A., Cluj-Napoca, Romania). From this day onwards, oral care with diluted chlorhexidine and borated glycerol was performed twice daily.

On day 5, the muscle rigidity remained unchanged and vocalization appeared. The phenobarbital regimen was reduced from 6 mg/kg intramuscularly every 12 h to 5 mg/kg intramuscularly once daily. Methadone 0.5 mg/kg once daily and propofol titrated to effect were also administered.

On day 7, an inflammatory reaction was observed at the insertion site of the central catheter, which was therefore removed. Limb massage with a comfrey root (*Symphyti radix*) ointment (Balsam cu Tataneasa, 30 mL; Quantum Pharm S.R.L., Cluj-Napoca, Romania) was started twice daily and continued until discharge.

Following general clinical reassessment, on day 10 of hospitalization a favorable evolution of the general condition was observed, the dog being conscious and consuming wet food and water voluntarily, although with difficulty. The nasogastric tube was removed. Treatment at this stage consisted of lactated Ringer solution as a constant rate infusion (5 mL/kg/h), Duphalyte 100 mL, levetiracetam 40 mg/kg every 12 h, amoxicillin with clavulanic acid 20 mg/kg every 12 h, pantoprazole 1 mg/kg every 24 h, metronidazole 20 mg/kg every 12 h, maropitant 1 mg/kg, metamizole 25 mg/kg every 12 h, baclofen 0.7 mg/kg before food every 8 h, Aspatofort two ampoules in lactated Ringer solution, diazepam as required (0.1 mg/kg intravenously in two divided doses) and methadone 0.2 mg/kg intravenously every 5 h. The tetanus antitoxin and metronidazole were discontinued on this day, after 10 consecutive days of administration.

On day 13 of hospitalization multiple episodes of diarrhea occurred, and treatment was reduced to amoxicillin with clavulanic acid 20 mg/kg orally every 12 h, pantoprazole 1 mg/kg every 24 h, baclofen 0.7 mg/kg in food every 8 h and a probiotic preparation (Florentero; Candioli Pharma, Beinasco, Turin, Italy) 0.5 mL orally, with loperamide added on day 14. Limb massage with the comfrey root (*Symphyti radix*) ointment was continued twice daily and physiotherapy was started on day 14.

On day 16 of hospitalization the dog was conscious and normothermic, with a normal appetite for both dry and wet food and normal urination and defecation. Limb flexion and extension had improved, and muscle stiffness had decreased further. In view of the favorable response to physiotherapy, the dog was discharged with the following recommendations: massage of the limbs at least twice daily; amoxicillin with clavulanic acid 20 mg/kg orally twice daily for 10 days; baclofen 0.7 mg/kg twice daily for 5 days; and pantoprazole 1.4 mg/kg once daily for 10 days, given in the morning on an empty stomach 30 min before food.

A recheck examination and a further physiotherapy session were scheduled 8 days after discharge. No information on this reassessment was subsequently available, and the clinical course described here therefore ends at discharge.

Several aspects of the therapeutical protocol deviated from the summary of product characteristics of the products used, or from dosages commonly reported in dogs, and are reported here for transparency. The tetanus antitoxin was administered for 10 consecutive days, whereas the label specifies daily administration of the therapeutic dose for 2 to 4 days. The same product is authorized for dogs from 6 months of age, whereas the patient was 5.5 months old at admission. The clinical record did not document a predefined endpoint for antitoxin administration. Continuation beyond the labelled duration was an empirical decision by the attending clinical team in the context of severe generalized disease that continued to progress during the first days of hospitalization. Antitoxin administration was discontinued on day 10, when the first clear signs of clinical improvement were recorded. This extended duration is reported for transparency and should not be interpreted as a recommended therapeutic protocol. Gabapentin was administered at 41 mg/kg every 12 h, above the range usually reported in dogs. Metronidazole was administered at 20 mg/kg every 12 h and levetiracetam at 40 mg/kg every 12 h, both above the dosages most commonly reported in dogs. The levetiracetam dose and dosing interval represented an empirical choice by the attending clinical team, and no formal justification for this regimen was documented in the clinical record. Its continuation was reassessed according to the overall clinical evolution of rigidity and spasms rather than according to predefined criteria. No epileptic seizures were documented during hospitalization, and neither the tetanic spasms nor the use of levetiracetam and phenobarbital should be interpreted as evidence of epileptic activity. Levetiracetam was administered empirically as part of the multimodal management of rigidity and spasms, whereas phenobarbital was introduced primarily as an adjunctive sedative and spasm-control medication. Baclofen, tolperisone, pantoprazole and the pyridoxine and aspartic acid solution are authorized for human use only and were therefore administered off-label; baclofen, given at 0.7 mg/kg every 8 h, is only rarely reported as a therapeutic muscle relaxant in dogs. Dexamethasone and hydrocortisone were administered on day 1 mainly as premedication before the first intravenous dose of heterologous equine serum, in order to reduce the risk of an anaphylactic reaction, and in part on empirical grounds; hydrocortisone was repeated on day 3 empirically. No adverse reaction attributable to the repeated administration of heterologous equine serum, and no clinical sign attributable to the gabapentin dose, were observed during hospitalization.

## 3. Discussion

This case report illustrates a severe form of generalized tetanus in a young dog characterized by multifocal neurological deficits and prolonged recovery. The clinical findings were consistent with the known pathophysiological mechanism of tetanospasmin, which inhibits glycine and GABA release at inhibitory interneurons, resulting in persistent muscle contraction and hyperreflexia [1].

Neurolocalization in this patient indicated multifocal involvement, with hyperactive spinal reflexes, reduced responses to nociceptive stimulation and impaired postural reactions accompanying the generalized rigidity. This distribution is consistent with an effect of tetanospasmin on inhibitory interneurons at spinal level, with extension to the brainstem, and it explains why a single neurological examination on admission is insufficient to follow the disease.

Cranial nerve dysfunction, indicated in this patient by dysphagia and facial muscle abnormalities, has previously been described in severe generalized tetanus. This could be linked to extension of toxin activity to brainstem pathways [21].

Respiratory complications are considered major causes of mortality in tetanus due to laryngospasm, aspiration, and respiratory muscle rigidity [22]. Mechanical ventilation was not required in the present case, although oxygen therapy was administered on day 3 of hospitalization.

Early recognition and aggressive supportive care are essential for successful management of canine tetanus. Multimodal therapy comprising sedation, muscle relaxation, antimicrobial therapy, nutritional support, physiotherapy and intensive nursing care is considered critical for a favorable outcome, and was implemented for this dog [16,28].

Magnesium has been used as an adjunctive muscle relaxant in canine tetanus [29]. Ionised magnesium remained within the reference interval throughout hospitalization in the present patient (0.61 to 0.63 mmol/L) and magnesium was not administered, muscle relaxation being achieved with the combination of agents described above.

According to the severity classification proposed by Burkitt et al. [20] and subsequently applied in canine tetanus, our patient was clinically classified as grade III on admission, being non-ambulatory with generalized rigidity, muscle tremors and dysphagia, in the absence of documented autonomic dysfunction. Heart rate, cardiac rhythm and arterial blood pressure were not recorded on admission, so grade IV could not be formally excluded; cardiovascular monitoring performed during general anesthesia on day 10 revealed no abnormality. Heart rate was monitored intermittently during hospitalization. Transient tachycardia was observed in response to handling and to external stimuli, consistent with the marked hyperesthesia of the patient, but no sustained tachycardia, arrhythmia or blood pressure instability suggestive of autonomic dysfunction was documented at rest.

In the retrospective analysis of 42 dogs reported by Zitzl et al. [9], non-survivors were more frequently younger than two years and had a shorter duration of specific clinical signs before presentation, and young dogs with rapidly progressive severe generalized tetanus were considered to carry a guarded prognosis. Our canine patient was 5.5 months old, developed generalized signs within approximately 24 h and was clinically classified as grade III, and therefore combined several of these recognized negative prognostic indicators, yet it was discharged ambulatory after 16 days and never required mechanical ventilation. Severe generalized tetanus has also been reported in other very young animals, including a litter of puppies with suspected neonatal tetanus [33] and a young cat [34]. Table 4 summarizes these and other published reports of tetanus in juvenile animals, together with selected reports in which electromyography or advanced neurological characterization was performed. Reports were identified by searching PubMed and Google Scholar, with no start-date restriction (earliest indexed records to August 2026), using combinations of the terms “tetanus,” “canine,” “dog,” “feline,” and “cat,” and were selected if they described tetanus in an animal under 12 months of age or reported electromyographic or advanced neurological characterization.

Among the reports identified, the present case was distinctive in documenting antitoxin antibody status before treatment and in applying a published severity classification.

Serum obtained within the hour preceding administration of the first dose of tetanus antitoxin was reported as negative (−) for antibodies against *Clostridium tetani* neurotoxin using the laboratory’s semi-quantitative LFA reporting system. Although anti-tetanus serology has no diagnostic value in tetanus, which remains a clinical diagnosis and a negative result neither confirms nor excludes the disease, this finding indicates that the pre-treatment sample was negative for circulating antitoxin antibodies according to the assay reporting system. Dörfelt et al. [16] used an LFA for the semi-quantitative assessment of anti-tetanus antibodies in dogs, with results categorized as negative (−), +, ++, or +++. In that study, four of five dogs tested 36–149 days after recovery from generalized tetanus had negative (−) results, suggesting that even convalescent animals may fail to develop measurable humoral immunity following natural infection [16]. Similar observations have been reported in humans, where recovery from clinical tetanus may be associated with absent or only low concentrations of circulating antitoxin [38]. Although these reports describe the post-recovery period and are therefore not directly comparable with the acute pre-treatment sample in the present case, together they support the concept that natural tetanus may fail to induce a measurable humoral immune response. This phenomenon is biologically plausible because the minute amount of tetanospasmin required to produce clinical disease is generally insufficient to elicit a measurable humoral immune response, and the toxin rapidly binds to neuronal tissue, limiting its exposure to the immune system [1]. Accordingly, our finding is consistent with the current understanding of tetanus immunology and further supports the view that the relative resistance of dogs to tetanospasmin is not attributable to pre-existing circulating antitoxin. The laboratory report became available only on day 7 of hospitalization, therefore this result did not contribute to the initial diagnosis or therapeutic decisions.

Among the ancillary diagnostic investigations performed in this case, needle electromyography (EMG) provided the most objective evidence supporting the presumptive clinical diagnosis while further characterizing the underlying neuromuscular dysfunction. Although tetanus remains primarily a clinical diagnosis relying on the patient’s history and characteristic neurological findings, EMG represents a valuable adjunctive diagnostic tool as it provides objective evidence of neuromuscular dysfunctions and facilitates discrimination between other disorders. EMG has previously been documented in cat and dog tetanus cases and has proven particularly useful in patients with atypical presentations or for the specific purpose of differentiating peripheral nerve, muscle or spinal cord disorders [10,15,37]. In generalized tetanus, the clinical presentation is often sufficiently characteristic to permit a presumptive diagnosis. The main differential diagnosis is strychnine toxicosis, which likewise impairs glycinergic inhibition [37]. Nevertheless, EMG can provide objective evidence of neuromuscular dysfunction, increase the diagnostic confidence, and assist in excluding alternative neuromuscular disorders when the presentation is atypical or when additional diagnostic evidence is considered beneficial. EMG is of greatest diagnostic value in focal presentations and for excluding other neuromuscular disorders such as polymyositis, spinal disease, hypocalcemia or meningoencephalitis [8]. In the present case, EMG was deliberately postponed until day 10 of hospitalization because the patient’s severe muscle rigidity and clinical instability during the acute phase made general anesthesia inadvisable. Priority was therefore given to stabilization and intensive supportive care, and the examination was performed once the patient’s condition allowed anesthesia to be administered with an acceptable level of risk. Despite the delay and the initiation of treatment, EMG demonstrated widespread abnormalities involving both axial and appendicular musculature, including moderately increased insertional activity, abundant positive sharp waves, fibrillation potentials, and persistent motor unit activity. Unlike the feline case reported by [10], in which electromyographic abnormalities were confined to the affected pelvic limb muscles in localized tetanus, our canine patient exhibited diffuse abnormalities consistent with generalized disease. Abundant positive sharp waves and fibrillation potentials are markers of muscle membrane instability rather than the continuous motor unit activity most often emphasized in canine tetanus [15,37]. In the present patient these changes were interpreted as reflecting secondary muscle membrane instability of uncertain origin, most likely associated with prolonged muscle hyperactivity and recumbency. Although an accompanying myopathy could not be completely excluded, serum creatine kinase activity had declined from 1692.8 U/L on day 2 to 134.8 U/L on day 9, one day before the EMG examination, which argues against an actively progressing myopathy at the time of recording. Motor unit activity was recorded while the dog was under general anesthesia with isoflurane and therefore could not have been voluntary; its persistence under general anesthesia supports a central origin resulting from tetanospasmin-induced loss of inhibitory interneuron function. When interpreted in conjunction with the patient’s history, serial neurological examination, clinicopathological findings, and characteristic clinical progression, these electromyographic abnormalities provided additional objective evidence supporting the presumptive diagnosis of generalized tetanus. Although EMG is not required for the diagnosis of most cases of generalized tetanus, it may be particularly valuable in referral practice when the clinical presentation is atypical, when important differential diagnoses remain under consideration, or when objective characterization of neuromuscular dysfunction is desirable. Accordingly, EMG is best interpreted as an adjunct to, rather than a substitute for, comprehensive clinical assessment and serial neurological examinations.

Pathological spontaneous activity appeared more prominent in the proximal than in the distal muscles examined. To our knowledge such a distribution has not been described in canine tetanus, but the present recording does not allow it to be established as a true gradient. The examination was qualitative, performed at a single time point under general anesthesia and restricted to one hemibody, and differences in muscle mass, in needle position relative to the endplate zone and in the local effects of prolonged recumbency may all contribute to an apparent proximal predominance. The observation is therefore reported as a hypothesis to be tested in future series in which the same muscles are sampled bilaterally and the findings are quantified.

Metronidazole was administered at 40 mg/kg per day for 10 consecutive days, and metronidazole neurotoxicosis has been reported in dogs [39]. Metronidazole was preferred to penicillin, in line with evidence from human tetanus indicating lower mortality with metronidazole [40]. In addition, penicillin is a γ-aminobutyric acid antagonist and could theoretically potentiate the disinhibition produced by tetanospasmin, providing a further rationale for this choice [24]. A drug-induced contribution to the neurological picture was considered unlikely, because the neurological signs were detectable before treatment was started, consisted of generalized rigidity rather than the vestibular and cerebellar signs that characterize metronidazole toxicosis, and improved while the drug was still being administered.

Packed cell volume fell from 42.4% on day 1 to 31.5% on day 4, with a microhematocrit of 29%, a reticulocyte count of 0% and normocytic indices, and the laboratory reported a normocytic non-regenerative anemia with eosinopenia. A fall of this magnitude over three days is faster than can be explained by reduced erythropoiesis alone. Repeated diagnostic phlebotomy in a patient of 7.3 kg, hemodilution resulting from the constant rate infusion of lactated Ringer solution at 5 mL/kg/h, and the corticosteroids administered on days 1 and 3 are considered the most plausible contributors. No source of blood loss was identified, no transfusion was required, and the anemia was not associated with clinical deterioration. Total protein was below the reference interval on days 1 and 9 (5.1 and 4.64 g/dL, respectively) and albumin remained in the lower part of its interval throughout hospitalization. The factors considered above for the fall in packed cell volume, namely repeated diagnostic phlebotomy in a 7.3 kg patient, haemodilution from the constant rate infusion of lactated Ringer solution and reduced voluntary intake during the dysphagic phase, are the most plausible contributors. No plasma or colloid was administered and no clinical consequence was attributed to these values.

Venous blood gas analysis on admission showed a lactate concentration of 4.9 mmol/L and a total carbon dioxide of 16.7 mmol/L with a pCO2 of 25.9 mmHg at a normal pH, a pattern consistent with a compensated metabolic acidosis attributable to sustained muscle contraction. Both lactate and total carbon dioxide had normalized by day 2 and remained within normal limits thereafter. The low venous oxygen saturation recorded on day 2 coincided with the clinical deterioration and with the appearance of opisthotonus, and preceded the institution of oxygen therapy on day 3. All samples were venous, so no conclusion can be drawn regarding pulmonary gas exchange.

Dexamethasone and hydrocortisone were administered on day 1 mainly as premedication before the first intravenous dose of heterologous equine tetanus antitoxin, with the aim of reducing the risk of an anaphylactic reaction to the heterologous serum, and in part on empirical grounds; hydrocortisone was repeated on day 3 empirically. No hypersensitivity reaction was observed at any point during the ten days of antitoxin administration. There is no established evidence supporting corticosteroid treatment for the tetanus itself, and the repeated administration of hydrocortisone on day 3 is reported as part of the clinical management of this individual patient rather than as a therapeutic recommendation. Corticosteroid administration provides a coherent explanation for the eosinopenia, for the hyperglycemia of 127 mg/dL recorded on day 1 and, in part, for the non-regenerative character of the anemia. Basal cortisol measured on day 2, after these doses, was within the reference interval, which limits any inference about the stress axis in this patient.

Animal welfare remained a priority throughout the 16-day hospitalization of the canine patient that remained recumbent for most of this period. Analgesia was multimodal and included methadone, butorphanol and metamizole, and sedation with benzodiazepines, phenobarbital, acepromazine and, on one occasion, propofol was titrated to reduce the frequency and intensity of tetanic spasms rather than to achieve immobility. Nursing care comprised repositioning every 4 h, enteral feeding every 6 h, oral care twice daily, limb massage and, from day 14, physiotherapy. Pain and distress were assessed clinically rather than with a validated scoring system, which is a limitation of this case report. Continuation of treatment was reassessed daily against the patient response, and euthanasia was not considered necessary at any point because the patient remained conscious, normothermic and free of respiratory compromise requiring ventilation.

### 3.1. Clinical Recommendations for Diagnostic and Therapeutic Management

Overall, this case reinforces that even severe generalized tetanus in juvenile dogs can have a favorable outcome when early recognition is combined with intensive supportive care, serial neurological assessment, and individualized treatment.

Generalized canine tetanus remains principally a clinical diagnosis, and early recognition of the characteristic combination of progressive generalized rigidity, stimulus-sensitive muscle spasms, hyperreflexia, cranial nerve-related abnormalities, and a compatible wound history is essential. Ancillary diagnostic testing should be directed primarily toward excluding competing neurological, neuromuscular, metabolic, and toxic disorders and identifying systemic complications.

Serial neurological examinations may be particularly useful for documenting disease progression and subsequent improvement. Electrodiagnostic examination can provide supportive evidence of persistent involuntary motor unit activity; however, in severely affected and clinically unstable patients, the risks associated with anesthesia should be weighed against the expected diagnostic benefit. In the present patient, EMG was postponed until clinical stabilization permitted anesthesia with an acceptable level of risk.

Management of severe generalized tetanus requires intensive multimodal supportive care tailored to the individual patient. Major priorities include neutralization of circulating unbound toxin when appropriate, antimicrobial treatment, reduction in muscle rigidity and stimulus-induced spasms, adequate analgesia and sedation, maintenance of hydration and nutrition, prevention of complications associated with prolonged recumbency, and close monitoring for respiratory and autonomic complications. When voluntary food intake is impaired, early enteral nutritional support should be considered. Frequent repositioning, appropriate nursing care, and physiotherapy during recovery are also important components of management.

Because multiple medications were administered concurrently in the present case, several at doses or durations outside commonly reported protocols, the favorable outcome cannot establish the efficacy of any individual treatment. Therapeutic decisions should therefore be individualized according to disease severity, clinical response, adverse effects, and available evidence.

### 3.2. Limitations of the Case Report

This report is subject to several limitations. Baseline arterial blood pressure, continuous electrocardiographic monitoring, and resting heart rate were not documented at admission. Consequently, autonomic dysfunction could not be formally excluded, and classification as Grade IV tetanus cannot be ruled out retrospectively. Although subsequent monitoring did not reveal sustained autonomic abnormalities, the patient should therefore be considered as having been clinically classified as Grade III based on the information available at presentation.

The puncture wound contained no devitalized tissue and was therefore not surgically debrided and no anaerobic bacterial culture or molecular testing for *Clostridium tetani* was performed. However, microbiological confirmation is not routinely required for the diagnosis of tetanus, which is primarily clinical, and identification of *C. tetani* at the suspected wound site may be difficult [1,8]. In the present case, the diagnosis was based on the compatible wound history characteristic and progressive neurological presentation, exclusion of relevant differential diagnoses, supportive electrodiagnostic findings, and subsequent clinical course. The absence of microbiological or molecular confirmation should nevertheless be considered when interpreting this report.

Serum biochemistry was performed in two different laboratories using different analytical methods and different reference intervals, which limits direct comparison between sampling days.

The electromyographic examination was performed with an instrument and software intended for human patients, for which no canine reference database exists, and the recordings were therefore interpreted qualitatively. The examination was restricted to the muscles of a single hemibody because the dog was not repositioned under anesthesia, so a side-to-side comparison was not available. In addition, the examination was not designed prospectively for standardized semi-quantitative grading, and the archived recordings did not permit reliable retrospective distinction of spontaneous activity of endplate origin from fibrillation potentials and positive sharp waves. Furthermore, the muscle from which the archived representative trace was obtained could not be retrieved from the stored recording, precluding reliable attribution of a retrospective score to an individual muscle. The EMG was also performed at a single point, after initiation of treatment and under general anesthesia, which may have influenced the electrophysiological findings. Nevertheless, characteristic abnormalities remained readily detectable and were considered diagnostically valuable when interpreted in conjunction with the clinical picture and disease progression. The apparent predominance of pathological spontaneous activity in proximal compared with distal muscles was based on qualitative assessment rather than standardized semi-quantitative grading. Consequently, no firm conclusion regarding a proximal-to-distal distribution can be drawn from this single examination, and confirmation using prospectively standardized EMG protocols and quantitative or semi-quantitative assessment in additional dogs is required.

Responses to nociceptive stimulation were assessed clinically and were substantially confounded initially by marked generalized rigidity. Serial assessments were also performed while the patient was receiving multimodal analgesia and sedation, which were progressively reduced during hospitalization. Consequently, the more consistent responses to nociceptive stimulation observed during subsequent examinations may reflect both neurological improvement and changes in medication, and the relative contribution of these two effects cannot be separated retrospectively. These findings should therefore not be interpreted as evidence of initially impaired and subsequently restored pain perception.

An additional methodological limitation concerns anti-tetanus antibody testing. Although subsequent clarification from the diagnostic laboratory identified the method as a lateral-flow assay (LFA) with semi-quantitative reporting, the manufacturer, catalogue/reference number, assay-specific validation characteristics, quantitative cut-off and limit of detection could not be retrieved retrospectively. Consequently, the negative (−) pre-treatment result cannot be assigned a specific quantitative antibody concentration or analytical detection threshold.

Several treatments, including the duration of tetanus antitoxin administration and the administration protocols for gabapentin, baclofen and metronidazole, deviated from the summary of product characteristics, as detailed above. The favorable outcome of a single patient does not allow conclusions regarding the efficacy or safety of these modified therapeutic approaches.

Finally, no post-discharge follow-up information was available. The outcome reported here is therefore limited to the clinical status documented at discharge on day 16, and the time to complete neurological recovery, as well as the presence or absence of long-term residual deficits, could not be determined.

### 3.3. Future Perspectives and Research Directions

The apparent greater abundance of pathological spontaneous EMG activity in proximal compared with distal muscles in this patient is an exploratory observation that requires confirmation. Future prospective studies should use standardized electrodiagnostic protocols, bilateral muscle sampling, predefined proximal and distal muscle groups, and quantitative or semi-quantitative grading of spontaneous activity. Serial EMG examinations at defined stages of disease and recovery could further determine whether electrophysiological abnormalities correlate with clinical severity, disease progression, treatment response, or prognosis.

Larger multicenter studies are also needed to refine prognostic indicators in canine tetanus and to evaluate the relative contribution of individual therapeutic interventions within multimodal treatment protocols. Such studies may help establish more standardized approaches to severity assessment, monitoring, supportive care, and the timing and clinical utility of electrodiagnostic testing.

## 4. Conclusions

This report documents the diagnostic workflow used to establish the presumptive diagnosis of generalized tetanus, clinically classified as grade III based on the information available at presentation, in a 5.5-month-old dog. The case presents the complementary roles of history, serial neurological examinations, clinicopathological assessment, and needle electromyography in supporting clinical decision-making and patient management. In addition, it describes electromyographic abnormalities and documents the absence of detectable circulating antitoxin antibodies before passive immunization. Despite severe and rapidly progressive disease, clinical improvement was observed from day 10 and the dog was discharged ambulatory and eating voluntarily on day 16. The favorable outcome should be interpreted in the context of a single patient managed with a complex multimodal protocol and does not establish the efficacy or safety of individual therapeutic interventions or deviations from commonly reported treatment protocols.

## Figures and Tables

**Table 1:** Serum biochemistry findings during hospitalization.

Parameter (Reference Interval)	Day 1	Day 2	Day 9
ALT (8–57 U/L)	52 U/L	NP	19.1 U/L
AST (<30 U/L)	NP	58.6 U/L	21.7 U/L
GGT (1–10 U/L)	8 U/L	NP	3.6 U/L
LDH (<200 U/L)	NP	549.2 U/L	86.2 U/L
CK (30–100 U/L)	NP	1692.8 U/L	134.8 U/L
Total proteins (5.4–7.5 g/dL)	5.1 g/dL	5.8 g/dL	4.64 g/dL
Albumin (1.75–3.07 g/dL)	2.9 g/dL	2.24 g/dL	2.12 g/dL
γ-Globulins (0.22–0.7 g/dL)	NP	0.35 g/dL	0.53 g/dL
Haptoglobin (0.01–0.2 g/dL)	NP	0.109 g/dL	0.126 g/dL
Fibrinogen (100–250 mg/dL)	NP	295 mg/dL	NP
Amylase (270–1462 U/L)	488 U/L	NP	NP
Total cholesterol (115.6–253.7 mg/dL)	212.82 mg/dL	NP	NP
Calcium (8.7–11.8 mg/dL)	9.74 mg/dL	NP	NP
Phosphorus (2.9–6.2 mg/dL)	6.99 mg/dL	NP	NP
Urea/Creatinine Ratio (8.51–115.97)	44.42	NP	NP
ALP (11–101 U/L)	107 U/L	NP	NP

ALT, alanine aminotransferase; AST, aspartate aminotransferase; GGT, gamma-glutamyl transferase; LDH, lactate dehydrogenase; CK, creatine kinase; ALP, alkaline phosphatase; NP, not performed.

**Table 2:** Blood gas analysis.

Parameters	Day 1	Day 2	Day 4
pH	7.393	7.363	7.423
pCO2 (mmHg)	25.9	39.9	30.3
pO2 (mmHg)	52.9	32.5	52.7
SO2 (%)	80	37	83
Hct (%)	42	32	32
tHb (g/dL)	14.1	9.8	9.1
Na (mmol/L)	145.8	143.1	139.6
K (mmol/L)	4.38	4.20	4.08
Cl (mmol/L)	113.7	113.1	113.7
iCa (mmol/L)	1.34	1.34	1.32
iMg (mmol/L)	0.62	0.63	0.61
Glu (mg/dL)	127	95	98
Lac (mmol/L)	4.9	0.5	0.4
Creat (mg/dL)	0.9	0.6	0.7
BUN (mg/dL)	9	8	8
TCO2 (mmol/L)	16.7	24.1	20.9

pCO2, partial pressure of carbon dioxide; pO2, partial pressure of oxygen; SO2, oxygen saturation; Hct, haematocrit; tHb, total haemoglobin; iCa, ionised calcium; iMg, ionised magnesium; Glu, glucose; Lac, lactate; Creat, creatinine; BUN, blood urea nitrogen; TCO2, total carbon dioxide.

**Table 3:** Hematological findings during hospitalization.

Parameter (Reference Interval)	Day 1	Day 4
WBC (6–17 × 10^9^/L)	13.04	14.63
LYM (1–4.8 × 10^9^/L)	2.17	4.22
NEU (3–12 × 10^9^/L)	10	9.32
EOS (0.1–1 × 10^9^/L)	0	0.05
MON (0.2–1.5 × 10^9^/L)	0.86	1.03
BAS (0–0.5 × 10^9^/L)	0	0.01
RBC (5.5–8.5 × 10^12^/L)	6	4.40
HGB (12–18 g/dL)	14.1	10.2
Hct (37–55%)	42.36	31.46
PLT (200–500 × 10^9^/L)	434	287

WBC, white blood cells; LYM, lymphocytes; NEU, neutrophils; EOS, eosinophils; MON, monocytes; BAS, basophils; RBC, red blood cells; HGB, haemoglobin; Hct, haematocrit; PLT, platelets.

**Table 4:** Published reports of tetanus in juvenile dogs and cats and selected reports documenting electromyography or advanced neurological characterization, compared with our findings. Fields that could not be read in the source publication are indicated as not stated. Reports were included either because the animal was under 12 months of age at presentation (juvenile) or because needle electromyography (EMG) or advanced neurological characterization was performed; the criterion met is given for each report. Reports listed on the basis of the second criterion alone may therefore concern adult animals.

Reference	Species, Breed, Age	Form and Severity	Key Features	Outcome	Inclusion Criterion
[33]	Dogs, American Bully, 3 to 5 days	Generalized	Extensor rigidity, trismus, inability to suckle; neurotoxin identified by mouse bioassay in one puppy	Favorable	Juvenile
[35]	Dog, Labrador Retriever, 10 weeks	Generalized	Acute facial swelling with no identifiable wound; progressive extensor rigidity of all limbs	Treated successfully	Juvenile
[36]	Dog, Border Collie, 4 months	Generalized	Two-day history of spasms and risus sardonicus; coxofemoral luxation treated by femoral head and neck excision	Favorable	Juvenile
Present report	Dog, Miniature Schnauzer, 5.5 months	Generalized, clinically classified grade III	Seronegative for antitoxin before treatment; electromyographic abnormalities qualitatively more prominent proximally than distally	Marked clinical improvement; discharged ambulatory after 16 days.	Juvenile, EMG
[10]	Cat, 9 months	Localized	Onset two weeks after castration; electromyography with bilateral pathological spontaneous activity of the biceps femoris	Improvement over 20 days	Juvenile, EMG
[34]	Cat, Domestic Shorthair, 10 months	Generalized	Chronic infected wound with necrotizing fasciitis; limb amputation	Died within one week of onset	Juvenile
[37]	Dog	Focal	Diagnostic value of electromyography in focal canine tetanus	Not stated	EMG
[15]	Two cats	Presumed localized	Electromyographic and F wave changes, with implications for diagnosis and prognosis	Not stated	EMG
[21]	Dog, Labrador cross, 11 years	Generalized	Onset with cranial nerve signs and magnetic resonance imaging abnormalities	Full recovery	Advanced characterization

## Data Availability

The original contributions presented in this study are included in the article. Further inquiries can be directed to the corresponding authors.

## Abbreviations

The following abbreviations are used in this manuscript:

CRP	C-reactive protein
ELISA	enzyme-linked immunosorbent assay
EMG	electromyography
GABA	gamma-aminobutyric acid
IU	international units
TeNT	tetanus neurotoxin
VAMP	vesicle-associated membrane protein
SNARE	soluble N-ethylmaleimide-sensitive factor attachment protein receptor
LFA	lateral-flow assay
